# The cascading origin of the 2018 Kīlauea eruption and implications for future forecasting

**DOI:** 10.1038/s41467-020-19190-1

**Published:** 2020-11-06

**Authors:** M. R. Patrick, B. F. Houghton, K. R. Anderson, M. P. Poland, E. Montgomery-Brown, I. Johanson, W. Thelen, T. Elias

**Affiliations:** 1 U.S. Geological Survey, Hawaiian Volcano Observatory, 1266 Kamehameha Avenue, Suite A8, Hilo, HI 96720 USA; 2 Department of Earth Sciences, University of Hawaiʻi at Mānoa, 1680 East-West Road, Honolulu, HI 96816 USA; 3 U.S. Geological Survey, California Volcano Observatory, 350 N. Akron Rd., Mountain View, CA 94035 USA; 4 U.S. Geological Survey, Cascades Volcano Observatory, 1300 SE Cardinal Court, Vancouver, WA 98683 USA

**Keywords:** Natural hazards, Volcanology

## Abstract

The 2018 summit and flank eruption of Kīlauea Volcano was one of the largest volcanic events in Hawaiʻi in 200 years. Data suggest that a backup in the magma plumbing system at the long-lived Puʻu ʻŌʻō eruption site caused widespread pressurization in the volcano, driving magma into the lower flank. The eruption evolved, and its impact expanded, as a sequence of cascading events, allowing relatively minor changes at Puʻu ʻŌʻō to cause major destruction and historic changes across the volcano. Eruption forecasting is inherently challenging in cascading scenarios where magmatic systems may prime gradually and trigger on small events.

## Introduction

**Fig. 1:**
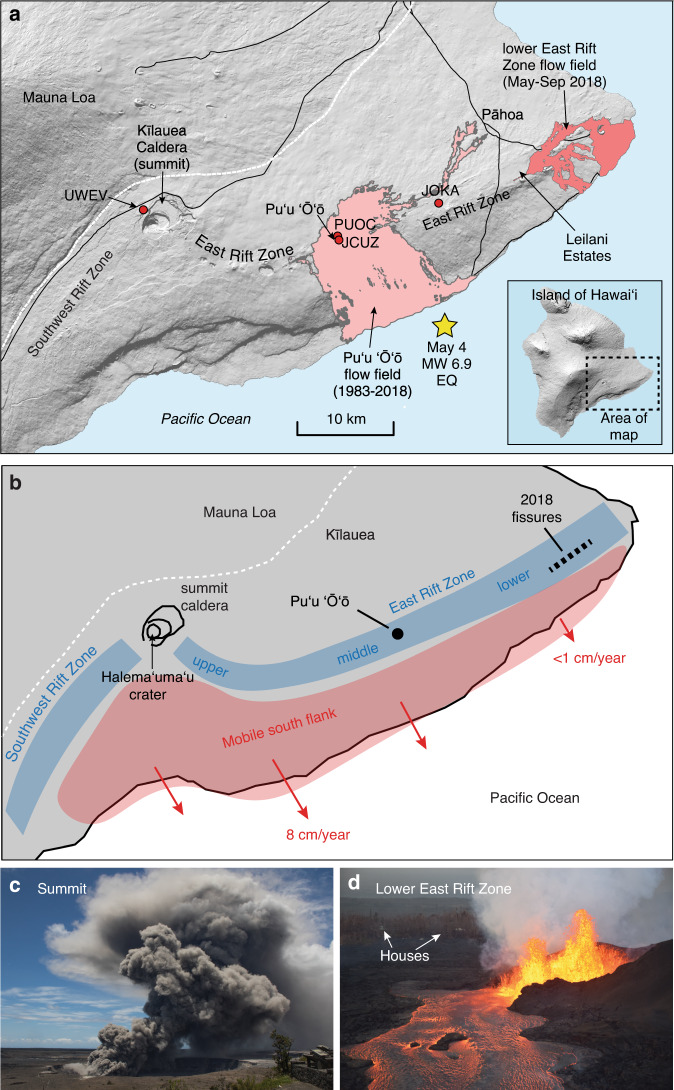
Location map for Kīlauea volcano. **a** Map of Kīlauea Volcano, on the Island of Hawaiʻi. The Puʻu ʻŌʻō eruption (1983–2018) produced a 144 km^2^ lava flow field in the middle East Rift Zone (ERZ). The May–September 2018 eruption occurred on the lower ERZ, ~40 km from the summit caldera. A large portion of the summit caldera floor subsided during the 2018 eruption. UWEV, PUOC, JCUZ, and JOKA are continuous GPS stations. The star shows the epicenter of the May 4 M_w_ 6.9 earthquake. **b** Schematic structural map of Kīlauea Volcano, showing the summit region and two rift zones. The mobile south flank exhibits steady southeast motion, and is tightly coupled with the rift zone magmatic system. **c** Small ash-rich explosive event at the summit, during the collapse of Halemaʻumaʻu crater, on May 15, 2018. USGS photo. **d** Fountaining (~50 m high) at fissure 8, the dominant vent in the lower East Rift Zone on June 5, 2018. Residences in Leilani Estates subdivision are visible in the background. USGS photo.

Kīlauea’s 2018 flank eruption, on the volcano’s lower East Rift Zone (LERZ) (Fig. 1), produced approximately one cubic kilometer of lava and was the most destructive volcanic event in the past 200 years in Hawaiʻi, with over 700 structures destroyed^1,2^. The accompanying collapse of the summit caldera was one of the largest at Kīlauea in centuries, triggering small explosions (Fig. 1c) and tens of thousands of earthquakes that damaged nearby infrastructure^1,3^. Kīlauea has one of the most comprehensive volcano monitoring networks on Earth^4^, and the 2018 eruption provides an excellent opportunity to understand the complex processes that culminate in destructive flank eruptions, the timescales of priming and triggering in open-vent basaltic systems, and how forecasting such extreme events might be improved.

Prior to 2018, the most recent eruptions from the LERZ occurred in 1955, 1960, and 1961^5^, and in 2014–2015 lava from the long-lived Puʻu ʻŌʻō eruption in the middle ERZ (MERZ) reached the outskirts of Pāhoa^6^ (Fig. 1a). Based on this historical activity and geologic mapping, volcanologists have long known that the LERZ is at high risk for lava inundation^7,8^. Likewise, several large collapses of the summit caldera floor have occurred over the past 200 years, primarily during the 1800s^9^. Despite the recognition of long-term hazard in these areas, short-term eruption forecasting must address factors of immediate relevance to hazard mitigation and is largely predicated on interpretation of geophysical and geological monitoring data^10^.

In this perspective, we characterize the changes at Kīlauea in the weeks to years leading up to the 2018 eruption. We highlight that the short-term cause was an increase in magma pressure due to a backup of magma in the shallow plumbing system, which ultimately drove magma into the lower flank of the volcano. Several processes, however, likely primed the magmatic system for years prior to the eruption. A cascading series of events caused a relatively small change at Kīlauea’s long-term ERZ eruptive vent to lead to historic consequences at the summit and a major destructive effusive eruption on the lower flank. The 2018 activity highlights the challenges in forecasting the form and timing of low-likelihood, large-volume eruptions that result from a cascade of interconnected processes.

## Forecasting volcanic eruptions

Forecasting volcanic eruptions remains fundamentally challenging, despite ongoing improvements in our ability to measure and understand the processes that prime and drive eruptive activity^10–13^. Once unrest is detected, volcano observatories must provide actionable assessments to emergency managers and the public that include the likelihood and potential timing of an eruption, its location, and ideally its scale and style. Even with robust monitoring networks and a strong understanding of a volcano’s geological and historical activity, these questions usually cannot be answered with confidence^11^. Magmatic systems are highly complex and cannot be directly observed^14^; therefore, volcanologists infer subsurface processes from often sparse monitoring data and idealized models. In addition, while magmatic systems may recharge and prime over extended intervals, the events that ultimately trigger an eruption occur over much shorter timescales—for example, the collapse of the north flank of Mount St Helens in 1980^15^. These limitations create significant uncertainty in forecasting scenarios.

Explosive eruptions may exemplify the common picture of volcanic hazards, but effusive (lava-producing) events, typical at basaltic systems, can also be dangerous and destructive. In human terms, the stakes of forecasting can be particularly high when lava effusion occurs low on a volcano’s flank, where population centers are common^16,17^. Eruptions at Nyiragongo (DR Congo) in 1977 and 2002, Etna (Italy) in 1669 and 2001–2002, Piton de la Fournaise (Réunion) in 1977, Mauna Loa in 1926, 1950, and 1984, and Kīlauea in 1960 and 1983–2018 are part of the long historical record of the risk to society posed by flank effusions^6,18–25^ and emphasize the critical importance of accurate forecasts during these events.

## Kīlauea Volcano and the 2018 eruption

Kīlauea’s magma originates in the mantle and rises into a reservoir complex beneath the summit caldera at depths of 1–5 km^26,27^. The summit reservoir system supplies magma to summit vents and laterally to rift zones that radiate to the east and southwest. Eruptions can occur at the volcano’s summit, where lava lakes have been common over the past 200 years, and/or along the rift zones (Fig. 1a, b); Kīlauea’s East Rift Zone has been especially active since the 1950s^9^. Magma can also be stored within the rift zones^28,29^, but the volume and geometry of this storage remains uncertain, especially below ~3 km depth^27^. Further complicating the picture, Kīlauea’s south flank moves seaward at rates of up to ~8 cm a year (Fig. 1b), imparting extensional stresses on the rift zones and facilitating rift zone magma transport, dike intrusions, and fissure eruptions^30–34^. Likewise, magma injected into the rift zones can produce stresses that trigger south flank motion and earthquakes^33–37^. Thus, the tectonics of the volcano’s south flank are tightly coupled with the magmatic system of the ERZ^27,38,39^.

Prior to 2018, the most recent eruptions in the LERZ occurred in 1955, 1960, and 1961 with eruptions focused on the MERZ and summit during the 1960s and 1970s^9^. Kīlauea erupted nearly continuously at or near the Puʻu ʻŌʻō eruption site in the MERZ (Fig. 1a, b) from 1983 until the onset of the 2018 LERZ eruption. For most of that time, slow-moving lava flowed south to the ocean, producing a 144 km^2^ flow field^25,40,41^. In 2008, a lava lake formed in Halemaʻumaʻu crater^42,43^ (Fig. 1b), at the volcano’s summit 20 km uprift from Puʻu ʻŌʻō, and persisted until the start of the 2018 eruption^1,3^.

### The 2018 eruption

Following several weeks of pronounced pressurization of the magmatic system at both the ERZ and summit eruptive vents, a small, brief fissure eruption occurred on the west flank of the Puʻu ʻŌʻō cone on April 30, 2018^1^. Over the next few days, earthquakes migrated eastward into the LERZ and rift-normal displacements were recorded by GPS instruments, signaling large-scale injection of magma downrift of Puʻu ʻŌʻō. Magma reached the surface in Leilani Estates subdivision on May 3, marking the onset of the LERZ eruption (Fig. 1a). The next day, a M_w_ 6.9 earthquake occurred on Kīlauea’s south flank (Fig. 1a)—the largest earthquake in Hawaiʻi in 43 years. The earthquake involved southward displacement of the mobile flank and is thought to be a consequence of stress induced by the ERZ injection^37^. Throughout May, 24 short-lived fissures developed in the LERZ, but activity focused on fissure 8 by the end of that month (Fig. 1d). The fissure 8 lava flow reached the ocean at the eastern tip of the island in early June, destroying several subdivisions and establishing a stable lava channel that persisted for two months^44^.

**Fig. 2:**
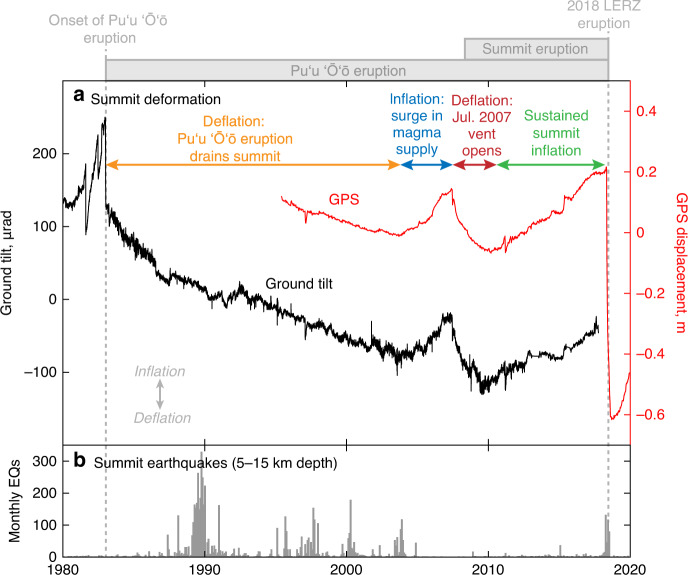
Deformation and seismicity at Kīlauea’s summit, 1980–2020. **a** Summit deformation (UWT radial ground tilt and and UWEV northward GPS displacement) showing deflation of the summit reservoir following the onset of the Puʻu ʻŌʻō eruption, interrupted by several years of inflation due to a surge in magma supply from the mantle. From 2010 to early 2018, the summit experienced sustained inflation, terminated by the 2018 lower East Rift Zone (LERZ) eruption. **b** Located deep crustal earthquakes (magnitude 1.7 and greater) beneath the summit (5–15 km depth), showing lower crustal swarms in the 1980s and 1990s that were not associated with changes in eruptive activity.

The LERZ eruption drained magma from the summit reservoir, 40 km away, at rates exceeding 100 m^3^ s^−1^, causing rapid summit deflation^3^. By mid-May, the summit lava lake had drained and the floor of Halemaʻumaʻu crater disintegrated in a piece-meal fashion, accompanied by several small explosive events (Fig. 1c). Summit collapses eventually involved larger portions of the caldera floor in June–July, with episodic piston-like failures that released energy equivalent to M5.2–5.4 earthquakes at intervals of 20–50 h. Significant lava effusion on the LERZ ended on August 4, roughly coincident with the end of summit collapse, although minor activity continued sporadically within the LERZ eruptive vent for the next month. Since September 2018 there has been no eruptive activity at Kīlauea, although ongoing inflationary ground deformation (Fig. 2a) and a subsurface mass increase since late 2018 indicate that magma is refilling the summit and ERZ^45^.

## Changes preceding the 2018 eruption

**Fig. 3:**
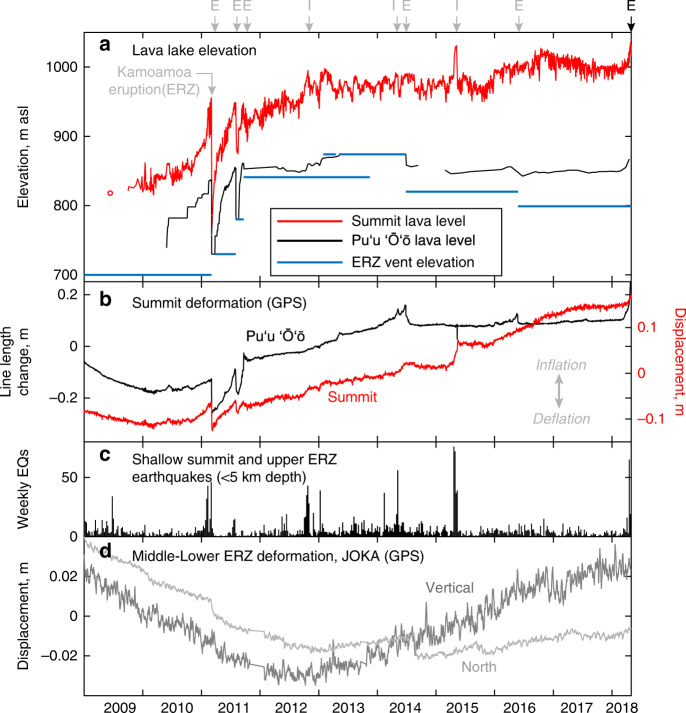
Long-term changes on Kīlauea, 2009–2018. **a** Elevation of the lava lakes at the summit and Puʻu ʻŌʻō, as well as the elevation of the vents at or near Puʻu ʻŌʻō. “E” notes times of eruptive vents forming on the East Rift Zone (ERZ) (at or near Puʻu ʻŌʻō), and “I” notes the time of intrusions at the summit—most eruptions and intrusions were preceded by rapid summit inflation and lava lake rise and an increase in shallow summit and upper ERZ earthquakes. The Kamoamoa eruption is noted specifically due to the broadly similar precursors it shared with the 2018 eruption. **b** Northward displacement of summit GPS station UWEV, and line-length change between Puʻu ʻŌʻō stations PUOC and JCUZ, showing a long-term inflationary trend at both eruption sites. **c** Shallow (<5 km depth) summit and upper ERZ earthquakes (magnitude 1.7 and greater), which often increase in rate during summit pressurization. **d** Displacement of GPS station JOKA, in the middle-lower ERZ, showing the onset of uplift in early 2013.

Kīlauea’s summit magma reservoir complex deflated for two decades following the onset of the Puʻu ʻŌʻō eruption in 1983 (Fig. 2a) as magma drained from the summit to supply the eruption^9^, then inflated from ~2003–2007 in response to a surge in magma supply^46^. Sustained deflation returned to the summit with the opening of a new vent near Puʻu ʻŌʻō in July 2007^40,47^. Inflation recommenced in late 2010 and was followed by a brief deflationary episode due to the formation of a new vent near Puʻu ʻŌʻō in March 2011^40^. Inflation continued into 2012 and subsequent years, likely caused in part by an increase in ERZ vent elevation^48,49^, and was accompanied by a net rise in the Halemaʻumaʻu lava lake (a proxy for magma reservoir pressure)^50,51^ (Fig. 3a). Inflation and lava lake rise rates at the summit increased in 2016 but leveled off in 2017^48^.

Geodetic data provide evidence for episodic magma transport downrift of Puʻu ʻŌʻō and into the LERZ during the years prior to 2018. Leveling and GPS data suggest a pause in long-term LERZ subsidence at the eastern tip of the island during 2003–2007, perhaps due to the arrival of magma in the LERZ linked to a surge in magma supply to Kīlauea^46^. Beginning in 2013, pauses in subsidence, and in some cases uplift, were observed at campaign and continuous GPS stations downrift of Puʻu ʻŌʻō (for example, at JOKA; Figs. 1a, 3d). These changes lasted until the 2018 eruption. The early 2013 change in deformation style at GPS stations downrift of Puʻu ʻŌʻō roughly coincided with changes in the summit and Puʻu ʻŌʻō eruptions. In January 2013, the Halemaʻumaʻu lava lake rose 50 m over 10 days, and the Puʻu ʻŌʻō lava lake rose several tens of meters, to one of the highest levels of lava in the crater in years^48,52^.

South flank motion (Fig. 1b) continued at a steady rate in the years prior to the 2018 eruption. Displacement rates were greatest (~8 cm yr^−1^) in coastal areas southeast of the summit, with rates diminishing to <1 cm yr^−1^ south of the LERZ^30^. Transient displacements occurred at semi-regular intervals during slow slip of the south flank of the volcano^38,53^.

**Fig. 4:**
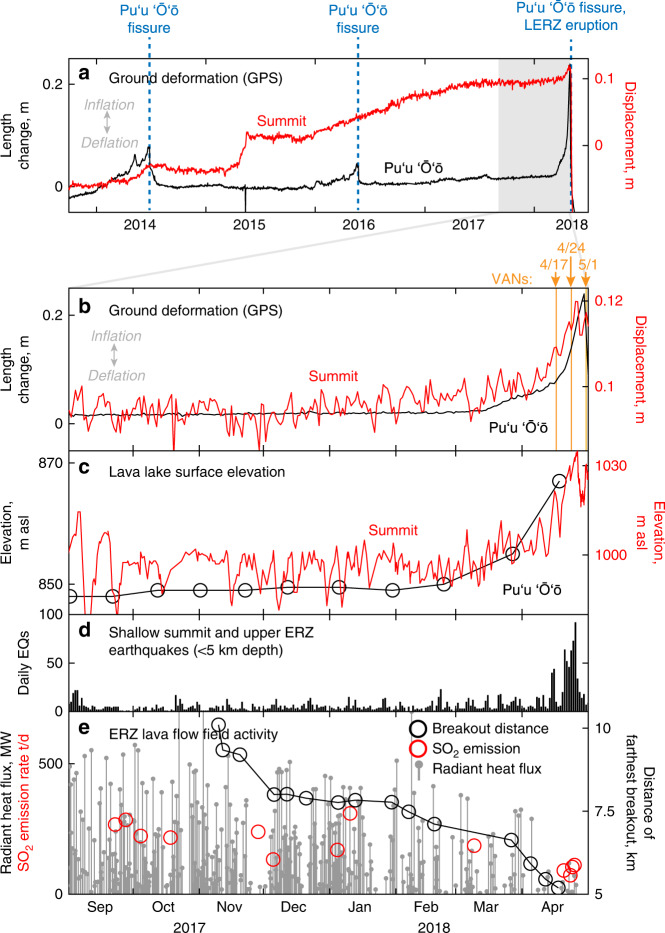
Short-term changes preceding the 2018 eruption. **a** Displacement of summit and Puʻu ʻŌʻō GPS, 2013–2018 showing the 2014, 2016, and 2018 vent openings. The 2014 and 2016 vents opened on Puʻu ʻŌʻō, while in 2018 a minor vent opened on Puʻu ʻŌʻō but was followed by a larger eruption on the lower East Rift Zone (LERZ). **b** Displacement of summit and Puʻu ʻŌʻō GPS from September 2017 to April 30, 2018, showing inflationary changes starting in March 2018. “VANs” shows the dates of Volcanic Activity Notices issued by HVO; the April 17 VAN noted the ongoing pressurization and forecast that a new vent could form on the East Rift Zone (ERZ), while the May 1 VAN noted magma moving east of Puʻu ʻŌʻō and forecast that a vent could form downrift. **c** Surface elevation of the lava lakes at the summit and Puʻu ʻŌʻō, showing an abrupt rise in March–April. **d** Shallow (<5 km depth) summit and upper ERZ earthquakes, which commonly increase in rate during summit pressurization. **e** Indicators of flow activity on the Puʻu ʻŌʻō flow field. “Breakout distance” shows the distance of the farthest surface lava breakouts from the Puʻu ʻŌʻō vent, measured along-tube, and shows a gradual retreat of breakouts upslope from November 2017 to April 2018. MODVOLC radiant heat flux, an indicator of surface flow activity, also decreased by April. SO_2_ emissions from Puʻu ʻŌʻō exhibited unusually low values in April.

**Fig. 5:**
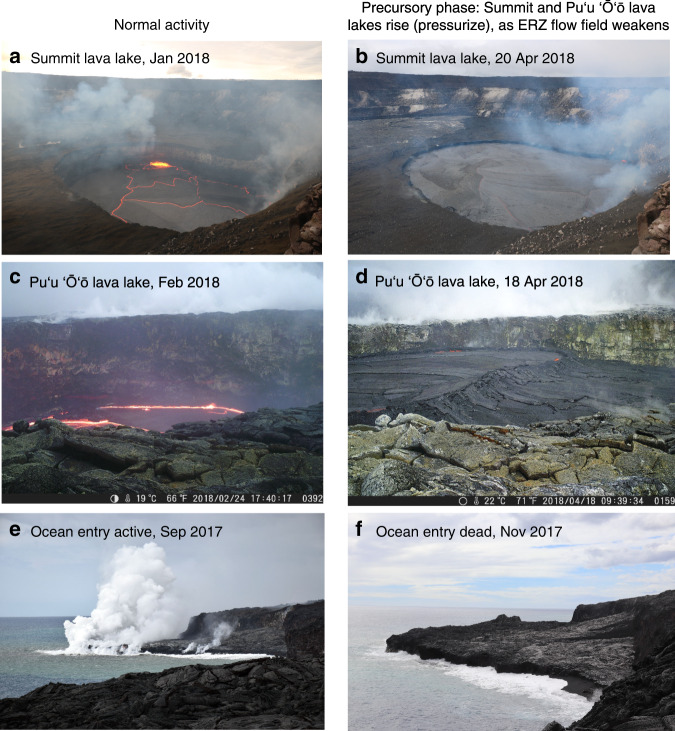
Visual observations of changes preceding the 2018 eruption. **a** Normal summit lava lake levels vs. **b** unusually high lake levels due to pressurization in April 2018. The lake is ~300 m long. **c** Normal lava lake elevation in Puʻu ʻŌʻō vs. **d** unusually high lake elevation due to pressurization in April 2018. The lake is ~50 m long. **e** Ocean entry activity typical of the preceding year, with numerous lava streams and a moderate steam plume vs. **f** an inactive ocean entry in November 2017 as East Rift Zone (ERZ) surface breakouts retreated upslope.

Ground deformation data indicate that Kīlauea’s shallow magma system—from the summit to Puʻu ʻŌʻō—showed low rates of deformation during 2017 and the first two months of 2018, but high rates of pressurization were recorded starting in mid-March 2018 (Fig. 4a, b). Lava lakes at both the summit and Puʻu ʻŌʻō rose to unusually high levels during that time period (Figs. 4c, 5), confirming pressurization in the magma system^1,51,54^. The summit lava lake produced the largest overflows on the Halemaʻumaʻu crater floor observed during the 10 years of the summit eruption. The small lava lake in Puʻu ʻŌʻō (Figs. 4c, 5c) also rose to unusually high levels in April 2018, and the adjacent crater floor was lifted up by roughly 15 m. An increase in shallow (<5 km depth) earthquakes at the summit and upper ERZ also occurred in April (Fig. 4d); similar earthquakes have commonly been associated with summit pressurization^48,51^.

Field observations and thermal satellite data indicate that the eruption at Puʻu ʻŌʻō was waning during early 2018 (Fig. 4e), despite the pressurization in the summit-ERZ magmatic system (Fig. 4b). In September–October 2017 the flow of lava at the ocean entry weakened, and the ocean entry shut down in mid November (Fig. 5f). From this time onwards, lava breakouts showed diminishing reach from the vent (Fig. 4e). MODVOLC thermal satellite data (Fig. 4e) showed reduced radiant heat flux from the lava flow field^55^ during March and April, and SO_2_ emission rates from Puʻu ʻŌʻō, used as an indicator of lava eruption rates^56^, were unusually low in April (Fig. 4e). These observations are all consistent with a reduction in the eruption rate from Puʻu ʻŌʻō during this time.

The rate of lower crustal earthquakes beneath the summit (5–15 km depth) increased in November 2017 and remained elevated into early 2018 (Fig. 2b), with several swarms in March 2018^57^. These rates were higher than in the previous 10 years (Fig. 2b), during which time these earthquake swarms were commonly associated with brief summit deflation episodes.

## What caused the 2018 eruption?

### Long-term priming of the magmatic-tectonic system

Long-term, seaward sliding of Kīlauea’s south flank (Fig. 1b) may have gradually set the stage for the 2018 LERZ eruption. Sliding of the south flank causes a corresponding increase in extensional strain in the shallow rift zone (<3 km)^38^, which consequently reduces the magma overpressure required for downrift propagation of a magma-filled crack. Along the MERZ, where steady south flank motion amounts to several cm/yr, “passive” intrusions have been known to occur as magma from the MERZ conduit rises toward the surface in response to rift zone extension, for example, in 1997^31^ and 1999^32^. South flank slip rates are much lower in the LERZ than in the MERZ (Fig. 1b), suggesting that it might take decades for enough extension to accumulate along the rift to facilitate intrusions. Consistent with this idea, a ~50-year recurrence interval has been estimated for major flank slip and rift opening events over the past 200 years at Kīlauea^39^. By 2018, 57 years had passed since the previous LERZ eruption and 43 years since the last major south flank earthquake—thus, the LERZ may have been poised for failure. This scenario has similarities with the 2004–2005 eruption of Etna Volcano, which was triggered by extension resulting from long-term motion of the eastern flank of the volcano^58^. Likewise, the 2018 eruption of Ambrym (Vanuatu) was facilitated by tectonically induced extensional stresses that prompted magma flow into the rift zone^59^.

Intrusions and eruptions at Kīlauea are frequently preceded by increases in magma pressure^9,27^, and Kīlauea’s magma system was unusually pressurized before the onset of the 2018 eruption. Inflation at the summit began in 2010 and was sustained through 2016 (Figs. 2a, 3a). By 2018, tilt and GPS data suggested the system was at its highest level of pressurization in at least 20 years (Fig. 2a). Puʻu ʻŌʻō was likewise in a prolonged inflated state since 2010 (Fig. 3a, b). The highly pressurized magma system would have increased the likelihood of an intrusive event and provided a greater head to drive magma into the LERZ^1^.

In addition, data suggest that leakage of magma downrift of Puʻu ʻŌʻō was occurring by early 2013 (Fig. 3d). The unusually high lava column in Puʻu ʻŌʻō in January 2013 may have provided sufficient overpressure at depth to open or expand a pathway downrift of Puʻu ʻŌʻō, which enabled gradual magma migration during 2013–2018 (Fig. 3d). Could the additional magma flow and heat transfer downrift of Puʻu ʻŌʻō after 2013 have facilitated the 2018 injection of magma into the LERZ? Campaign GPS data collected annually since 1995 in the middle and lower ERZ suggest that at least one period of downrift magma transport occurred prior to 2007, with no accompanying eruption^46^. It is possible that periods of slow magma transport downrift of Puʻu ʻŌʻō have occurred regularly in the past, but this remains poorly understood.

### Weeks-long increase in magma pressure due to backup

Kīlauea’s magma system began to pressurize much more rapidly during March–April 2018, shown by inflation, rising lava lake levels, and increasing shallow summit and upper ERZ earthquakes (Fig. 4b–d)^1,60^. Increased magma pressure at Kīlauea is sometimes associated with higher eruption rates from ERZ vents^40,49,51^, while at other times it is associated with a decrease in eruption rates^40,61,62^. In the latter instances, as during early 2018 (Fig. 4e), pressurization may be explained as the result of reduced output at the Puʻu ʻŌʻō vent, causing magma to backup in the system^40^. The cause of the reduced output is not well understood but, like in 2018, previous instances of backups at Puʻu ʻŌʻō occurred after the vent persisted for several years^40,48,62^, uggesting that the shallowest conduit feeding the vent may tend to atrophy due to reduced transport efficiency over time. One strong possibility is that a restriction of some form develops in the shallowest conduit connecting the Puʻu ʻŌʻō reservoir to the vent feeding the lava flow field, inhibiting conduit flow. The efficient hydraulic connection between Puʻu ʻŌʻō and the summit results in magma accumulation throughout Kīlauea’s shallow magma system^48^. Historically, rapid inflation from this process has culminated in the formation of formation of new MERZ vents^40,48,61,62^.

Kīlauea’s shallow magma system is also known to pressurize in response to increases in magma supply from the deeper magmatic system, but we see no clear evidence for an increase in deep magma supply in early 2018. Changes in magma supply had been documented in the mid-2000s and had a significant and direct impact on summit inflation and MERZ eruptive activity^46,49^. During early 2018, however, the MERZ eruption rate decreased, rather than an increase as might be expected from an increase in deeper magma supply^46^. Furthermore, there was no change in the character of the deeper portion of the magma reservoir complex (3–5-km depth), as had occurred during the mid-2000s^46^. CO_2_ emission rates, previously used as a proxy for deep magma supply rates^46^, were not available in the years immediately prior to 2018 due to challenging measurement geometry. There was an increase in lower crustal (5–15-km depth) earthquakes in late 2017 to early 2018 relative to the previous decade^57^ (Fig. 2b), but previous work has shown that these lower crustal earthquakes, common in the 1980s and 1990s, are not clearly related to changes in eruptive activity^63^, and their source mechanism remains ambiguous. In the context of activity that has occurred at Kīlauea since 2008, when the geometry of the magmatic system was most similar to early 2018, an increase in magma supply is not needed to explain the inflation and seismicity during that time.

### Eruption trigger

On April 30, 2018, a small intrusion occurred into the west flank of the Puʻu ʻŌʻō cone, similar to the culmination of previous episodes of rising pressure that created new vents on or around Puʻu ʻŌʻō^40,48,61,62^. This event, however, coincided with a larger injection of magma far downrift of Puʻu ʻŌʻō, creating the first large-scale magmatic episode in the LERZ in 57 years.

What changed in the plumbing system to allow large volumes of magma to enter the LERZ? A persistent feature must have existed in the rift zone that prevented significant downrift magma transport past Puʻu ʻŌʻō during the 35 years of magma supply to the vent. Localized barriers to magma transport have been previously hypothesized in the ERZ based on seismic data^64^, and for diking events at other volcanoes^65^. Multiphase mixture models suggest that a section of rift east of Puʻu ʻŌʻō was exceptionally dense^66^, perhaps making it difficult for new cracks to initiate or propagate downrift. Vents have opened slightly east of Puʻu ʻŌʻō several times during the eruption^47,61^, but were fed by very shallow dikes that probably emanated from the Puʻu ʻŌʻō feeder system, implying that any long-lived barrier to downrift magma transport was rooted deeper, in the main ERZ conduit. The 2013 and onwards deformation downrift of Puʻu ʻŌʻō (station JOKA; Figs. 1a, 3d) suggests that such a barrier may have been leaky, raising questions on how the feature may have evolved—or degraded—over three decades. The barrier must have been sufficiently resilient, however, to shunt the majority of magma to Puʻu ʻŌʻō despite numerous disruptions during the 35-year eruption. In addition, the entire magmatic pathway downrift of Puʻu ʻŌʻō, having been largely abandoned for decades, may have been so poorly developed as to permit nothing more than a trickle of magma prior to 2018.

Whether due to a localized barrier near Puʻu ʻŌʻō or to the intrinsic resistance to flow in the largely abandoned, vestigial pathway east of Puʻu ʻŌʻō, downrift flow might have been impeded prior to 2018 simply because magma overpressure was insufficient to initiate new cracks. The long-term pressurization of the system (Fig. 2a), coupled with the short-term perturbation of April 30, may have finally exceeded the threshold needed to overcome this resistance to flow. Flow into the LERZ was probably aided by long-term dilation of the rift zone due to south flank motion (Fig. 1b). The combination of rift dilation and magma pressurization may simply have reached a critical threshold by late April 2018.

There remain unanswered questions in this conceptual model that deserve further study, particularly with regard to the exact failure process that allowed magma to move east of Puʻu ʻŌʻō. Nonetheless, the onset of the 2018 eruption can be adequately explained by intrinsic magmatic and tectonic processes. Recent work has proposed that heavy rainfall triggered the 2018 eruption, based on a purported lack of significant precursory inflation^67^. The high rates of widespread inflation and lake level rise in the weeks prior to the 2018 eruption (Figs. 4b, c, 5), however, indicate that increasing magmatic pressure was the dominant driver, and extrinsic triggers such as rainfall are not required to explain the eruption.

## The historic scale of the 2018 eruption

The conceptual model above explains the buildup to the 2018 activity at Kīlauea, but why was the eruption so large? Previous work has demonstrated that eruptions along Kīlauea’s LERZ tend to be infrequent but relatively large (0.1–0.3 km^3^ in 1790, 1840, 1955, 1960^5,68^). This is likely due to the lower elevation of LERZ vents, which require lower overpressures to drive flow, and can drain magma storage zones more thoroughly (including magma stored in the ERZ). Indeed, for 18 ERZ eruptions in the 20th century, summit deflation (a proxy for pressure change) scaled inversely with vent elevation^69^. The volume of the 2018 eruption (approximately one cubic kilometer), however, was large even by LERZ standards, so other factors, noted below, must also have contributed.

An open question regards the role of the May 4 M_w_ 6.9 south flank earthquake in influencing the magnitude of the LERZ eruption. The timing of the earthquake, days after the onset of magma moving into the LERZ^70^, suggests that the intrusion triggered the earthquake by stressing the south flank^37^, as proposed for previous episodes where rift zone intrusions apparently induced strong south flank earthquakes^47^. Did the M_w_ 6.9 earthquake dilate the ERZ and enable higher rates of magma transport? An apparent increase in the rate of summit drainage after the earthquake supports the notion that the M_w_ 6.9 earthquake boosted transport rates in the magmatic system^3^. If the M_w_ 6.9 earthquake did enhance magma transport to the LERZ, this might, in part, explain the comparatively large erupted volume that triggered structural failure at the summit^3^. In comparison, the 1955 and 1960 LERZ eruptions occurred in a similar area of the rift zone but were much smaller (0.1–0.3 km^3^)^5,68^, were not associated with ≥M6 south flank earthquakes, and did not produce large-scale collapse at the summit.

The role of the collapsing caldera at the summit of Kīlauea in the evolution of the eruption also requires further study. Episodic failure of the rock above the summit magma reservoir renewed the pressurization of the reservoir with each collapse and produced transient increases in eruption rate; these events therefore probably played a role in sustaining the eruption^3,44,71^. A quasi-exponential decay of pressure in Kīlauea’s deeper summit magma system over the course of the eruption, however, suggests additional summit processes also affected the magnitude of the event.

## A cascading magmatic process

**Fig. 6:**
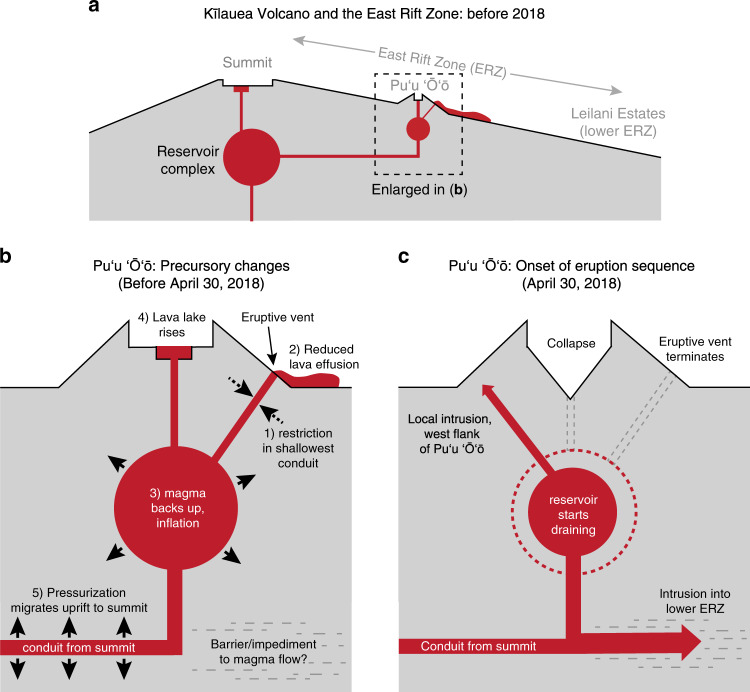
Schematic showing the changes leading to the 2018 eruption. **a** Cross-section of Kīlauea from the summit down the East Rift Zone (ERZ), prior to 2018. Two simultaneous eruptions were occurring (summit and Puʻu ʻŌʻō). **b** Proposed changes at Puʻu ʻŌʻō that led to the 2018 eruption. A restriction between the Puʻu ʻŌʻō magma reservoir and lava flow vent is hypothesized to have reduced lava flow effusion rate, causing magma to backup and accumulate in the magmatic system. This produced pressurization at Puʻu ʻŌʻō, and the summit via the ERZ magma conduit. **c** Onset of the 2018 eruption sequence at Puʻu ʻŌʻō. Overpressure produced a local intrusion on the west flank of Puʻu ʻŌʻō and initiated the larger injection of magma into the lower ERZ. Magma flow into LERZ triggered drainage of the Puʻu ʻŌʻō magma reservoir, causing crater floor collapse and termination of the lava flow vent.

How did relatively minor events at Puʻu ʻŌʻō (Fig. 6b) progress to the historic scale of the 2018 eruption? We propose that the 2018 eruption of Kīlauea began and evolved as a cascading series of events (Table 1), which was difficult to anticipate due to the complexity of the system^72,73^. Cascading sequences are intrinsic to volcanic eruptions and can occur over a wide range of spatial and temporal scales. In an idealized explosive eruption, for example, pressure in the reservoir drives magma towards the surface, and decreasing pressure eventually allows gas exsolution to occur. Bubble growth then enhances ascent rates and leads to fragmentation, producing an eruption^74^. The resulting eruption hazards may also occur in a cascading manner^75^.

**Table. 1:** Priming processes and cascading events of the 2018 eruption of Kīlauea.

Possible priming processes (long-term)		
	Process	Time period
a	Steady south flank motion accumulates extensional strain in lower East Rift Zone	decades preceding 2018
b	Prolonged inflation of summit magma reservoir	2010–2018
c	Slow leakage of magma into lower East Rift Zone	2013–2018, and earlier

Cascading event sequence (short-term)		
Steps	Event	Time period
1	Restriction in shallowest conduit feeding vent at Puʻu ʻŌʻō	late 2017 to April 2018
2	Reduction in lava flow output	late 2017 to April 2018
3	Magma backs up, plumbing system pressurizes, inflation from Puʻu ʻŌʻō to summit	March–April 2018
4	Pressurization reaches critical level, drives small intrusion at Puʻu ʻŌʻō and larger injection of magma into LERZ	30-Apr-2018
5	LERZ intrusion produces LERZ eruption	3-May-2018
6	LERZ intrusion triggers M_w_ 6.9 south flank earthquake	4-May-2018
7	M_w_6.9 earthquake dilates rift zone, facilitating greater magma transport (?)	May 4 to August, 2018
8	LERZ eruption drains magma from summit reservoir	May–August, 2018
9	Summit reservoir draining causes caldera floor collapse, frequent summit earthquakes	May–August, 2018
10	Caldera floor collapse triggers small explosions	mid-May 2018

**Fig. 7:**
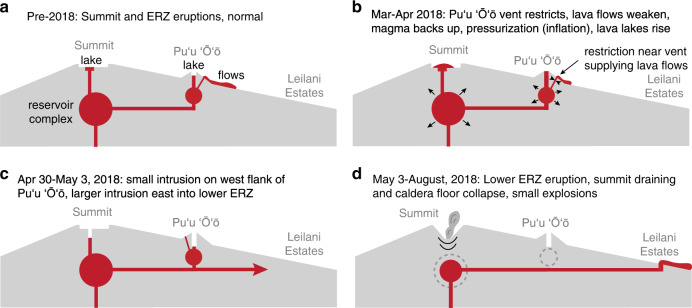
Overview of precursors and 2018 eruption stages. **a** Prior to 2018, summit and Puʻu ʻŌʻō eruptions were ongoing, and were jointly supplied magma from the summit reservoir complex. **b** During early 2018, a restriction near the Puʻu ʻŌʻō lava flow vent caused magma to backup, driving concurrent inflation at the summit and Puʻu ʻŌʻō, and rising lava lakes at both sites. **c** This pressurization reached a critical threshold on April 30, 2018, when a small intrusion occurred at Puʻu ʻŌʻō and a larger intrusion was initiated that migrated into the lower East Rift Zone. **d** The lower East Rift Zone (ERZ) intrusion created an eruption on the lower flank of the volcano, which led to substantial draining of the summit magma reservoir, driving caldera collapse, and small explosions.

In the 2018 eruption of Kīlauea the cascade sequence was a chain of events that was unforeseen at the onset of unrest (Table 1; Fig. 7). The magmatic system may have been primed for years due to (a) gradual dilation of the rift zone due to south flank motion, (b) the prolonged inflated state of the magmatic system, and/or (c) slow magma leakage into the LERZ. The cascade was set in motion in late 2017 and early 2018 as a restriction developed in the vent conduit supplying magma to the Puʻu ʻŌʻō lava flows (step 1), reducing lava outflow (step 2) and causing magma to backup and pressurize the system (step 3), opening a pathway and/or clearing a barrier near Puʻu ʻŌʻō that allowed a larger scale magma migration east into the LERZ (step 4). Magma reached the surface as a LERZ eruption (step 5). The input of magma into the LERZ imparted stress on Kīlauea’s south flank, which triggered the M_w_ 6.9 earthquake (step 6), relieving confining stress on the rift zone which, in turn, may have enhanced magma transport to the LERZ eruption site (step 7). The LERZ eruption removed magma at a high rate from the summit magma reservoir (step 8), causing collapse of the caldera floor (step 9) that led to small explosions (step 10) and maintained magma reservoir pressure, in part sustaining the eruption. This convoluted sequence links a relatively small change near Puʻu ʻŌʻō to major, destructive lava effusion on the LERZ (20 km downrift) and historic changes at the summit (20 km uprift), all enabled by an efficient hydraulic connection along the ERZ.

## Challenges in forecasting the 2018 activity

We can learn for the future by examining how Hawaiian Volcano Observatory (HVO) scientists assessed the activity as it was unfolding. The long-term inflation at the summit (Fig. 2a) and uplift east of Puʻu ʻŌʻō (Fig. 3d) (for example, at station JOKA, Fig. 1a), were recognized by HVO scientists as indicating system-wide inflation, but the gradual nature of the trends did not clearly point to short-term hazard.

The short-term precursors in March–April—pressurization at both the summit and Puʻu ʻŌʻō—were identified immediately (Fig. 4b–d). During this phase of pressurization, the consensus among HVO staff was that a new vent would most-likely form on or near Puʻu ʻŌʻō, following patterns in 2011, 2014, and 2016 (Figs. 3a, b, c, 4a). With the inflation persisting into April, HVO issued a Volcanic Activity Notice (VAN) on April 17 that stated “Observations…during the past month suggest that the magma system beneath Puʻu ʻŌʻō has become increasingly pressurized. If this activity continues, a new vent could form at any time, either on the Puʻu ʻŌʻō cone or along adjacent areas of the East Rift Zone.” At that time, a primary concern was that such a vent might appear on the north side of the Puʻu ʻŌʻō cone, sending lava into a catchment that could eventually reach populated areas, as happened during the 2014–2015 Pāhoa lava flow crisis^6^. A VAN issued on April 24 highlighted the increased pressurization and high level of the summit lava lake, and the possibility of a new vent forming on or near Puʻu ʻŌʻō.

The expected local intrusion occurred at Puʻu ʻŌʻō at approximately 14:20 HST on April 30, creating a brief fissure eruption and small flows on the west flank of the cone. What was not expected, however, was the continuation of earthquakes and further magma injection downrift of Puʻu ʻŌʻō that commenced within the subsequent hours. The earthquakes reached the area of Highway 130, 18 km east of Puʻu ʻŌʻō, by midday on May 1. This recorded the first major movement of magma into the LERZ since the 1960s and was an unambiguous signal that larger, and potentially more hazardous, changes were underway. On May 1 (04:54 HST), HVO issued a VAN alerting the public of the evolving hazard and stating that an outbreak of lava in a new location was one possible outcome. The focusing of earthquakes beneath Leilani Estates on May 2, and the opening of small ground cracks on that day, suggested that an eruption could occur in this area. On May 2 (19:23 HST) the status report was updated to indicate that an outbreak of lava from the LERZ remained a possible outcome of the continued unrest. The LERZ eruption began 21 h later, at ~17:00 HST on May 3.

The VANs and status reports released during the precursory phase in April did not forecast significant hazards at the summit, based on lack of recent precedent^40^. Previous intrusions in the area of Puʻu ʻŌʻō, such as in 2011, produced significant summit deflation but no large-scale structural changes to the caldera floor, nor summit explosive activity^40,48^. The 1955 and 1960 LERZ eruptions caused localized sagging and disintegration of the Halemaʻumaʻu crater floor^24,76^, but not large-scale caldera collapse or explosive activity. Once the 2018 LERZ eruption commenced and the Halemaʻumaʻu lava lake began rapidly draining, however, HVO recognized the possibility of explosive events^77^, similar to those in 1924^78^, which also followed lake draining and a LERZ intrusion. On May 9 a VAN stated that the dropping lava level “raised the potential for explosive eruptions in the coming weeks.” Relatively minor explosive activity began in mid-May and continued throughout the month.

## Lessons learned for future eruptions

The onset of Kīlauea’s 2018 eruption was forecast accurately in the weeks leading up to the event, but its location and size were not. What can volcanologists learn from these events when responding to future activity at Kīlauea and other volcanoes?

First, the 2018 eruption serves as a cautionary tale against over-reliance on recent volcanic activity as a guide for future behavior. Kīlauea’s Puʻu ʻŌʻō eruption had persisted for decades despite numerous perturbations of the magmatic system^25,32,40,46,47^ and appeared to be a testament to the stability of eruptive activity in the MERZ. Based on parallels with magma injections in 1991, 2011, 2014, and 2016, inflation in early 2018 suggested only a new MERZ intrusion or formation of a new vent at Puʻu ʻŌʻō—a fundamental change to the eruption was not expected. In retrospect, the March–April inflation and the sequence of events that was anticipated to result from it served as a point of focus and may have distracted from consideration of Kīlauea’s broader geologic record^5^, which includes four LERZ eruptions in the past 200 years^5^, one (1840) of which triggered collapse of the caldera floor. Several additional large collapses of the caldera floor occurred in the 1800s;^9^ however, unlike 2018, none of these previous LERZ events or summit collapses occurred in the midst of an ongoing, multiyear MERZ eruption^9^.

Humans may naturally focus on obvious changes and most-likely outcomes at the expense of less obvious changes and less likely outcomes. The predilection to see the future as similar to the immediate past can be considered a kind of tunnel vision^79^, which can have detrimental effects on unbiased, comprehensive consideration of information^80^ and illustrates a challenge of forecasting volcanic eruptions using short- to intermediate-term pattern recognition^10^. The risks of tunnel vision may be alleviated in part by considering the broader geologic history of a volcano, which can serve as a useful reminder that other (possibly much larger) outcomes are also possible, even if unlikely^10^. These possible outcomes must be considered during each new phase of evolving unrest, even if a previously recognized pattern appears to be repeating itself.

Evident precursors to the 2018 eruption were relatively small and provided a deceptive underestimate of the scale of the impending eruption. Thus, the 2018 eruption highlights the challenge of forecasting complex cascading sequences of events. At Kīlauea, the extensive rift zone magmatic system has previously exhibited complex interactions with the summit reservoir. In 1924, an intrusion in the LERZ withdrew magma from the summit reservoir and caused the floor of Halemaʻumaʻu crater to collapse, followed by a series of summit explosions^78^. At Stromboli Volcano, increasing magma pressure at the volcano’s summit has previously triggered flank eruptions, leading to rapid decompression of the deeper reservoir and dangerous paroxysmal explosions^81,82^. The 1980 Mount St. Helens eruption began with a M5.1 earthquake and landslide of the unstable, bulging flank, which then exposed a cryptodome of magma and removed overburden from the central conduit, resulting in a lateral blast and Plinian explosion^15^. In each of these examples, major aspects of the eruption could not be anticipated in a straightforward manner from the immediate signs of unrest. Whether in a cascading sequence or not, unforeseen volcanic events are common from a global perspective^10^, reinforcing the need for implementing forecasting frameworks that account for remote outcomes.

It may never be possible to determine if or when a particular event, such as a small magma injection, might trigger a sequence of cascading events and culminate in a large eruption. Volcanic systems are highly nonlinear and behave chaotically and unpredictably^83–85^; however, small events can only trigger large events if the state of the volcanic system—such as the volume and pressure of eruptible magma and tectonic stress state—permits it^86^. An important focus of future work should thus be to better understand when systems may be primed such that a small trigger can result in a large eruption. These conditions may be characterized using monitoring data together with conceptual and mathematical models, and interpreted in light of geological and historical records, which can be used to make inferences on the types and recurrence rates of future activity.

Quantitative hazard forecasting tools, such as probabilistic event trees^87–89^ and Bayesian belief networks^90–92^, allow scientists to rigorously integrate information from geologic mapping, monitoring data, models, and even expert opinion, to obtain probabilistic assessments of possible future activity. In some cases, these tools can be used to obtain not only a forecast of future activity, but also quantitative insight into the state of the volcanic system (e.g., whether or not magma is ascending^91^). Also, importantly, the utilization of these tools requires careful analysis and discussion of possible outcomes and may thereby reduce the tendency towards tunnel vision. The simple act of carefully discussing possible outcomes may bring a greater awareness of the possibility of low-probability high-impact events and help observatory scientists consider a broader range of outcomes. It should also encourage vigilance for the prospect that ostensibly small changes at a volcano could, given the right circumstances, evolve into much larger and more hazardous activity. We also advocate for observatory scientists to become familiar with these tools—and to use them to develop basic long-term forecasts that can be modified as needed—well before the onset of a volcanic crisis.

Finally, we emphasize that volcanologists must remain humble no matter how sophisticated our data and models become. Volcanoes often erupt in unexpected ways^10^. Stromboli, for instance, has been studied for centuries, and the onset of flank effusion is now recognized by Italy’s volcano monitoring agency as a possible precursor to paroxysmal explosions^81,82^—an outstanding example of eruption forecasting based on monitoring data, the historical record, and an understanding of the volcanic system. Nonetheless, two paroxysmal explosions in 2019 (one fatal) occurred in the absence of flank activity^93^. As with Kīlauea’s 2018 eruption, these events highlight the limits of current understanding even at relatively well-studied volcanic systems.

## Conclusions

In this perspective, we propose a conceptual model for the processes that led to the historic 2018 eruption at Kīlauea Volcano. Monitoring data indicate that a backup in the magma plumbing system at Puʻu ʻŌʻō drove pressurization, which opened a pathway for magma into the LERZ. Open questions remain on the failure process near Puʻu ʻŌʻō that allowed magma to migrate into the LERZ and how Kīlauea’s LERZ and broader magmatic system might have been primed prior to 2018. The eruption evolved in a cascading manner that was difficult to forecast, resulting in an eruption that was greater in magnitude and impact than expected based on previous analogous events. Cascading events present an inherent challenge to eruption forecasting by producing outcomes that may be unanticipated, or deemed unlikely, based on the immediate precursors. A greater awareness of this issue, and more focused research on cascading events and the processes that prime them, should better prepare volcano observatory scientists for future eruptions.

## Methods

### Geodetic, seismic, and gas emission monitoring

Ground deformation was monitored with a network of GPS instruments and tiltmeters^27,94^. Changes at the summit are shown by the northward displacement of station UWEV (Fig. 1a). At Puʻu ʻŌʻō, deformation is shown by line-length changes between stations PUOC and JCUZ (Fig. 1a), situated on opposite sides of the cone. Station JOKA (Fig. 1a) is used to show changes in East Rift Zone deformation east of Puʻu ʻŌʻō.

Seismicity is monitored with a network of short-period, strong-motion and broadband seismometers^95^. Due to requirements of solar power and real-time telemetry, seismometers along the East Rift Zone are generally concentrated along the topographic axis. The density of stations prior to the 2018 eruption falls significantly downrift of Puʻu ʻŌʻō, with only four continuous seismometers between Puʻu ʻŌʻō and Cape Kumukahi. The sparsity of stations and linear geometry leads to a host of artifacts in earthquake locations^96^. Locations uprift of Puʻu ʻŌʻō to the Kīlauea summit do not have those artifacts in general. Seismicity plots (Figs. 2b, 3c, 4d) show earthquakes located by HVO’s seismic monitoring network. Sulfur dioxide emissions (Fig. 4e) were measured with an ultraviolet spectrometer used in vehicle-based traverses beneath the Puʻu ʻŌʻō gas plume^56,97,98^.

### Geologic monitoring

The lava lakes at Halemaʻumaʻu and Puʻu ʻŌʻō were monitored with a combination of visual observations, laser rangefinder measurements, and webcam and timelapse camera images^48^. The level of lava in the Halemaʻumaʻu lava lake was tracked with daily laser rangefinder measurements (Fig. 4c)^43^. The Puʻu ʻŌʻō lava lake was monitored with a timelapse camera, with lake surface height measurements (Fig. 4c) made sporadically with a laser rangefinder, referenced to a benchmark measured with kinematic GPS. The Puʻu ʻŌʻō lava flow field was monitored with helicopter overflights every few weeks, with the position of surface breakouts tracked with handheld GPS as well as airborne thermal mapping and satellite imagery^40,41,52,99,100^. The lower flow field and ocean entry were monitored during ground visits as well^77^. A timelapse camera was also used to monitor the ocean entry activity. Lava flow field surface activity was also tracked with the MODVOLC satellite-based estimates of radiant heat flux (Fig. 4e) (http://modis.higp.hawaii.edu/)^55^.

## Data Availability

Seismic data are available at IRIS (iris.edu). GPS data are available at UNAVCO (unavco.org). SO_2_ data are previously published^98^. The lava lake elevation data analyzed in this study will be made available on ScienceBase (sciencebase.gov).
